# Effects of whole body vibration in postmenopausal osteopenic women on bone mineral density, muscle strength, postural control and quality of life: the T-bone randomized trial

**DOI:** 10.1007/s00421-022-05010-5

**Published:** 2022-07-21

**Authors:** Yvonne Kienberger, Robert Sassmann, Florian Rieder, Tim Johansson, Helmut Kässmann, Christian Pirich, Anton Wicker, Josef Niebauer

**Affiliations:** 1 University Institute of Physical Medicine and Rehabilitation, Paracelsus Medical University, Salzburg, Austria; 2 Institute of General Practice, Family Medicine and Preventive Medicine, Paracelsus Medical University, Salzburg, Austria; 3 University Institute of Nuclear Medicine and Endocrinology, Paracelsus Medical University, Salzburg, Austria; 4 University Institute of Sports Medicine, Prevention and Rehabilitation, Paracelsus Medical University, Salzburg, Austria

**Keywords:** Osteopenia, Postmenopausal, Whole body vibration training, Resistance training, Bone mineral density, T-score

## Abstract

**Purpose:**

Osteopenia is common in postmenopausal women and effective interventions increasing or stabilizing bone mineral density (BMD) to prevent fractures are urgently needed.

**Methods:**

Sixty-five postmenopausal women diagnosed with osteopenia (T-score between -1.0 and -2.5) were randomly assigned to either a vibration training group (VT), a resistance training group (RT), or a control group (CG). BMD T-score values (primary endpoint) were assessed at baseline (T0) and after 12 months (T12), secondary endpoints (muscle strength, postural control, and health-related quality of life) at baseline (T0), after 6 months (T6), after 12 months (T12), and as follow-up after 15 months (T15).

**Results:**

After the intervention period, neither the VT nor the RT showed any significant changes in BMD T-score values compared to the CG. Isokinetic strength improved significantly within all training groups, with the exception of the flexors of VT at an angular velocity of 240°/s. Health-related quality of life as well as postural control improved significantly for the RT only.

**Conclusions:**

We conclude that participants of all three groups were able to maintain their BMD. The improvements in quality of life and postural control after resistance training are nevertheless meaningful for postmenopausal osteopenic women and support the importance of regular loadings of the musculoskeletal system.

This study was retrospectively registered in January 2022 at the DRKS (S00027816) as clinical trial.

**Supplementary Information:**

The online version contains supplementary material available at 10.1007/s00421-022-05010-5.

## Background

Osteoporotic fractures are a major individual and socioeconomical burden in our aging society (Burge et al. 2007). Therefore, prevention of osteoporosis is essential. Osteopenia is considered a precursor to osteoporosis and is characterized by T-score values between − 1.0 and − 2.5. At this stage at the latest, interventions to increase or maintain bone mineral density (BMD) are justified. 

Resistance training positively affects both BMD (Howe et al. 2011) and muscle strength (Macaluso and De Vito 2004). However, patients’ adherence and compliance with this lifelong training intervention is less than optimal. Strenuous exercises, generating high-intensity loading forces to the skeleton (Marques et al. 2012), can be challenging and require regular, at least weekly visits to training facilities. Alternative, less-demanding and time-efficient training regimes are therefore of utmost importance for a good prognosis.

Whole body vibration training (WBVT) is a promising intervention that increases muscle strength (Machado et al. 2010) and balance (Ritzmann et al. 2014). In fact, enhanced markers of bone formation (Chung et al. 2014; Xie et al. 2006) and bone density (Wenger et al. 2010) have been reported in animal studies. There is conflicting evidence in humans. While some authors found increases in BMD after WBVT (e.g., Zha et al. 2012; Iwamoto et al. 2005; Verschueren et al, 2004; Von Stengel et al. 2011a, b), others reported no effect (e.g., Liphardt et al. 2015; Slatkovska et al. 2011). In addition, previous meta-analysis were not able to clarify the effect of WBVT on BMD (Lau et al. 2011; Oliveira et al. 2016;). One explanation might be the large heterogeneity in study designs, delivery of intervention (e.g., intensity, length of intervention), and WBVT specifications ranging from 12 to 113 Hz, 0.2 to 32.2 g or 0.005 to 8 mm amplitude (Lau et al. 2011). A previously published meta-analysis (Fratini et al. 2016) suggests that training in a static squatting position on a side-alternating vibration platform with accelerations ≥ 3 g is the most effective WBVT protocol to enhance BMD in the hip and lumbar spine in postmenopausal women. However, when these settings were used in osteopenic women, no beneficial effects of WBVT were found on BMD in the lumbar spine or the femoral neck, bone microarchitecture of the distal radius and the tibia, or balance and strength (Liphardt et al. 2015). Of note, the participants performed the same WBVT static squatting settings during the 12 month intervention period. For this long period of time, load progression might be necessary to provide further physiological adaptation stimuli. In addition, BMD for the total hip was not reported by Liphardt et al. (2015). However, this whole area has been shown to be specifically stimulated using side altering vibration platforms (Rittweger 2010). Therefore, some regional effects of WBVT on BMD need further evaluation.

### Aims

Hence, the primary objective of this study was to investigate the effects of progressive WBVT and resistance training in postmenopausal osteopenic women on BMD T-score values of the total hip and lumbar spine. Secondary objectives were effects of WBVT and resistance training on strength, balance, and quality of life. Both interventions were compared to a control group (CG).

## Methods

### Study design

This study was designed as a single-blinded, randomized controlled trial including three study groups: a vibration training group (VT), a resistance training group (RT), and a control group (CG). The intervention period lasted for 12 months and included two training sessions per week. A follow-up examination was performed 3 months after the end of the active intervention period. Patients were randomly allocated by using sealed opaque envelopes (*n* = 90) of a 1:1:1 ratio of VT, RT, and CG. All physicians remained blinded to the patient’s allocation. Due to the nature of the intervention, participants could not be blinded. Patient identity was independently coded after enrollment and decoded after data analysis.

### Participants

Eligible participants were postmenopausal women aged ≥ 45. Postmenopausal was defined as 12 consecutive months without menstruation. Exclusion criteria included: acute disc prolapse, acute thrombosis, acute inflammation of the musculoskeletal system, kidney or bladder stones, acute hernias, epilepsy, seizures, pregnancy, arrhythmias, pacemaker, osteoporotic-induced fractures, multiple sclerosis, chronic obstructive pulmonary disease, uncontrolled angina pectoris, coronary heart disease, uncontrolled cardiac failure, high-grade aortic stenosis, hypertrophic cardiomyopathy, dizziness, surgery, or hospitalization within the previous 6 months and receiving any drug treatment for osteoporosis affecting bone metabolism and/or muscle strength within the last year.

### Recruitment

Eligible participants were identified at the University Institute of Nuclear Medicine and Endocrinology, Paracelsus Medical University, Salzburg. BMD T-score values were determined by dual-energy X-ray absorptiometry (DXA- QDR 4500 W, Hologic, Marlborough, Mass., USA) on the left side of the hip and at the lumbar spine (L1-L4). Subjects were diagnosed as osteopenic when T-score values between − 1.0 and − 2.5 were measured at least at one of these sites. Patients, who met inclusion and none of the exclusion criteria, were invited to participate in the trial. All patients were fully informed about the research purpose, possible adverse events but also expected health benefits. Recruitment took place from February 2010 to June 2012.

### Intervention programs

Two sport scientists at the Institute of Physical Medicine and Rehabilitation supervised the intervention from March 2010 to December 2013. Standardized training programs (vibration and resistance training group) were applied as described below.

### Vibration training

The VT group performed different exercises standing barefoot on a side-alternating vibration platform (Galileo Sport, Typ 8N056001C, Pforzheim, Germany) twice a week for 12 months. The exercises were introduced gradually, depending on the physical condition of the patients. During the first 3 weeks, patients began static squats with a knee angle of about 30° (0° = full extension) three times for 1 min (frequency (*f*): 18 Hz, amplitude (*A*): 2 mm) with 1 min rest between repetitions, increasing to 6 repetitions (f: 20 Hz, A: 2 mm) of 1 min each, corresponding to approximately 3.2 G (calculated with the equation *g* = *A*(2π*f*)^2^/9.81). After 3 weeks of static squatting and reaching the target frequency, load progression was ensured by adding dynamic upper body exercises (e.g., by lifting dumbbells or using swing sticks) during the static squat. In this phase, patients performed three static squats of 1 min each without and three static squats with upper body exercises on the vibration plate. If this was well tolerated by the patients, additional dynamic squats were added to the program. Finally, one training session included 2 bouts of static squats without upper body movement, 2 bouts of static squats with different upper body exercises, and 2 bouts of dynamic squats (supplemental material 1). Exercises were added for patients only if the present program could be performed without problems (for example balance). Before vibration, patients began their exercise session with a 10-min warm-up on a bicycle ergometer (Daum electronic, Fürth, Germany) at 1.0 W/kg and a cadence of 50–70 per minute and ended it after vibration with a cool-down, including stretching of the leg, hip, and lumbar muscles. In total, the entire training lasted about 30 min.

### Resistance training

Patients in the RT group trained according to the Austrian recommendations for health-promoting activity for (older) adults, consisting of 10–15 repetitions to the point of volitional fatigue, 2–3 series of all large muscle groups (Titze et al. 2010). Resistance exercise training was performed twice a week for 12 months at the Institute of Physical Medicine and Rehabilitation, University Hospital Salzburg, Austria under the supervision of sport scientists. Each training session lasted for about 45 min in total. Resistance training started with a 10 min warm-up on a cycle (Daum electronic, ergo bike Medical8, Fürth, Germany) or upper body ergometer (Proxomed, kardiomed upper body cycle, Typ 2011, Alzenau, Germany) followed by balance exercises for another 10–15 min, e.g., on wobble boards. Afterward, resistance training was performed on 6 different machines: leg press, leg abduction, leg flexion, leg extension, latissimus machine, and pulley. During the first 12 weeks of resistance training, the intensity was systematically increased from three sets of 20 repetition of 50–60% one repetition maximum (RM) to three sets of 10–15 repetitions of 70% 1RM (supplemental material 1). Weights were noted for each individual patient and adjusted every 2 weeks.

### Control group

Subjects of the CG were instructed to maintain their current level of physical activity during the study period and not to engage in any new form of exercise.

### Data assessment and acquisition

**Fig. 1:**
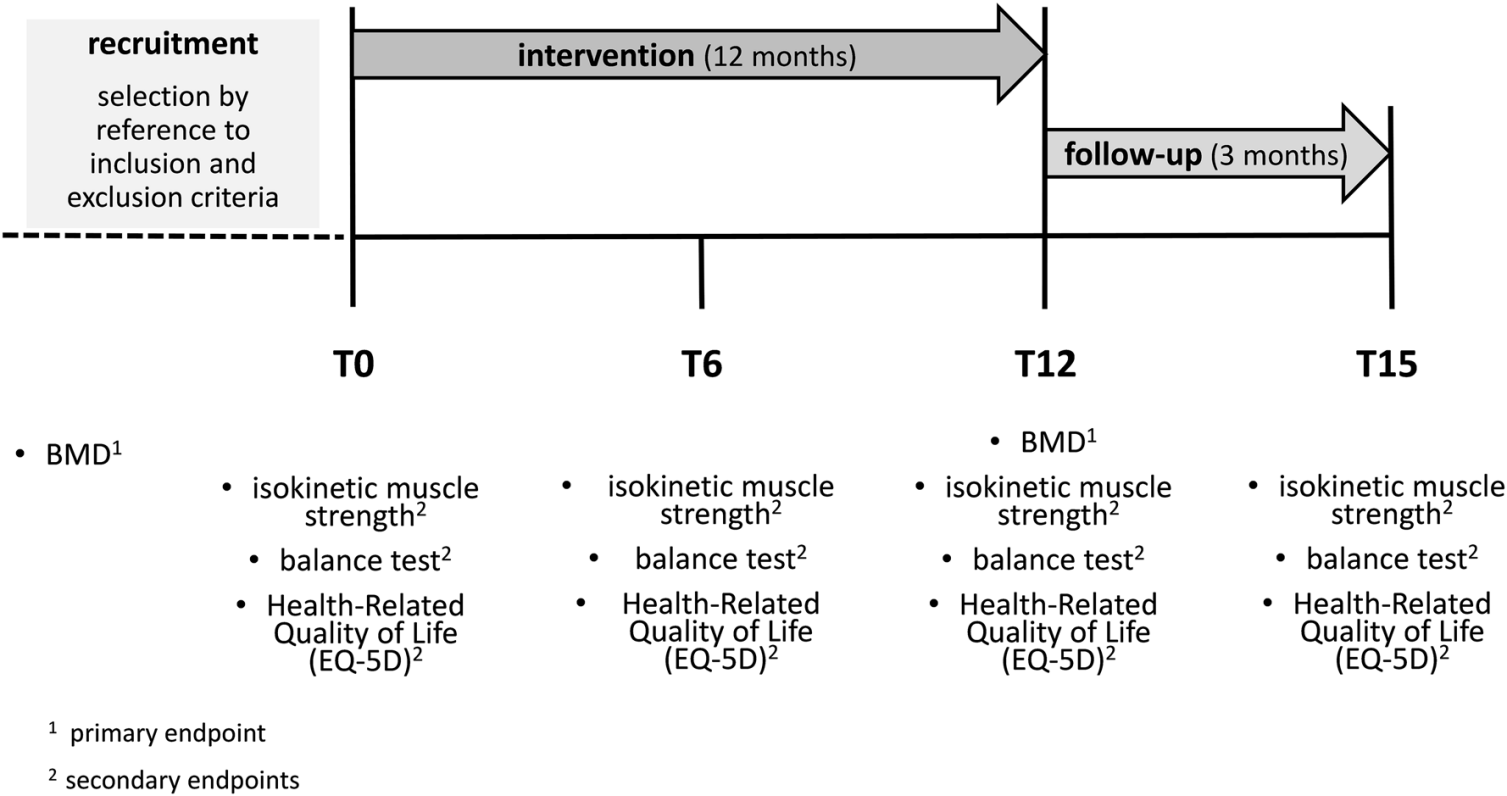
Timeline

BMD T-score values were measured at baseline (T0) and after 12 months (T12). Isokinetic strength, balance, and quality of life were assessed at baseline (T0), 6 (T6), 12 (T12), and 15 (T15) months (Fig. 1).

### Primary outcome

At baseline (T0) and 12 months (T12), BMD was determined by DXA on the left side of the hip and the lumbar spine (L1–L4) (Tothill and Hannan 2007). Coefficients of variation have previously been reported as 1.3% for the lumbar spine and 1.4% for the femoral neck region (Von Stengel et al. 2011a, b). All scans were done by an experienced technician, who was blinded to the patients´ group allocation. An overview of the data collection and outcome assessment is presented in Fig. 1.

### Secondary outcomes

#### Isokinetic muscle strength assessment

The isokinetic strength of the participants’ left knee extensors and flexors was measured on an isokinetic dynamometer (BIODEX System 3 and 4Pro, Biodex Medical Systems, USA) at baseline (T0), 6 (T6), 12 (T12), and 15 (T15) months (Fig. 1). The test–retest-reliability of this method has already been shown by Feiring et al. (1990) (knee extension peak torque at 60 degrees/sec (°/s), *r* = 0.95 and at 240°/s, *r* = 0.95). The test was done at two different angular velocities 60°/s with 5 repetitions (reps) and 240°/s with 25 reps. With these two test speeds, on the one hand, the maximum torque during a slow muscle contraction (60°/s) and thus approximately 66% of the maximum muscular power was determined and on the other hand, the faster muscle contraction (240°/s), which from a functional point of view corresponds to the speed of movement of the knee joint during human gait at 4 km/h. Patients were allowed to have 3–5 trial repetitions at each angular velocity directly before the outcome assessment. Between the different angular velocities, patients had a 90-s resting period. Relative peak torque to body mass (DMM/kg) at each testing speed was used to determine knee extension and flexion strength.

#### Postural control assessment

Postural control was measured by the “GGT” balance test (GGT: Acronym for the German word “Gleichgewichtstest”, i.e., balance test in English) (Bös et al. 1992). Quality criteria of this postural assessment were followed (Wydra 1993). This assessment consisted of 14 different items that determine static and dynamic balance ability. Each item consists of exercises with increasing difficulty. The more exercises one completes per item, the higher the score. The balance test was performed at baseline (T0), 6 (T6), 12 (T12), and 15 (T15) months (Fig. 1).

#### Health-related quality of life

The EQ-5D-3L questionnaire, self-administrated by the participants, was used to evaluate health-related quality of life (EuroQolGroup, 1990). The validity and reliability of the EQ-5D has been assessed for the different language versions and various health conditions (Van Hout et al. 2012). EQ-5D-3L index (EQ Index) and EQ visual analogue scale (EQ-VAS) were analyzed according to the manual. The questionnaire was completed at baseline (T0) and after 6 (T6), 12 (T12), and 15 (T15) months (Fig. 1).

### Statistical analysis

All statistical analyses were performed with IBM SPSS Statistics 23.0. Figures were produced using the GraphPad Prism 9.0.0 (GraphPad Software Inc., La Jolla, United States). We evaluated our primary endpoint in an intention-to-treat analysis according to the CONSORT guidelines for the reporting of randomized controlled trials (Schulz et al. 2010). We applied the method of Baseline Observation Carried Forward (BOCF) for missing data with respect to the primary endpoint (T-score). A per-protocol analysis was performed for all secondary endpoints. To be included into the per-protocol analysis, the training compliance needed to be 80% or higher, achieved by 16 women (76.2%) in the VT and 19 women (79.2%) in the RT. All tests were two-tailed, and a 5% probability level was considered significant. The Shapiro–Wilk test was used to check for normal distribution. Homogeneity of variance was determined using Levine’s *F* test. The intervention effects on primary and secondary endpoints were tested using the Kruskal–Wallis test to compare the delta values (baseline – follow-up, mean differences) of the groups. Within-group differences in pre-post-analysis were tested using the *t* test for paired samples (primary endpoint). The Friedman test was used analyzing within-group differences of all secondary endpoints. A post hoc Dunn–Bonferroni test was calculated for parameters with significant group difference. For comparison of baseline characteristics of the three groups, a one-way ANOVA was used for normally distributed variables.

## Results

### Participants, recruitment, and process evaluation

**Fig. 2:**
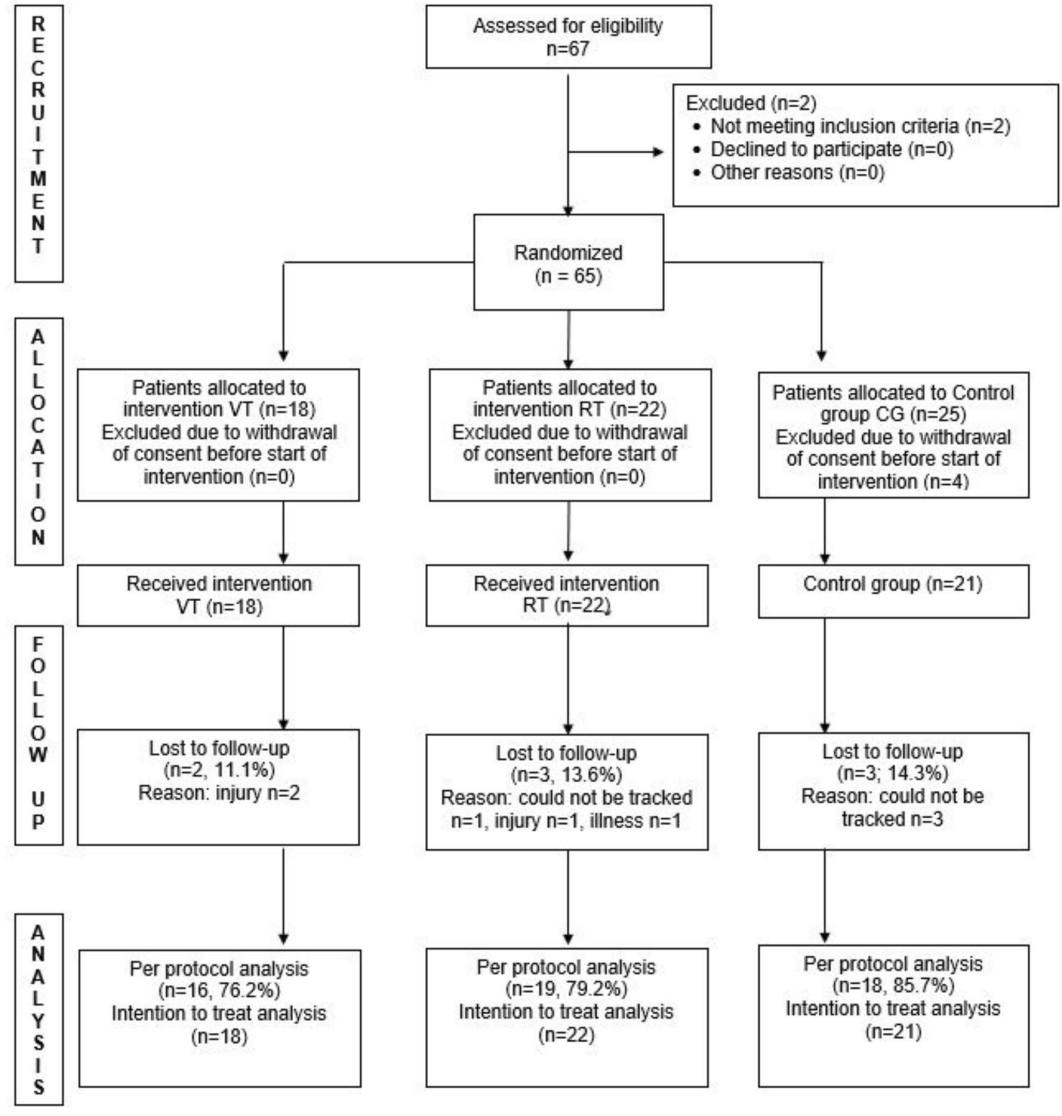
Consort chart illustrating participants recruitment and retention

A total of 67 eligible patients signed informed consent. Two patients did not meet inclusion criteria; therefore, 65 patients were randomly allocated to one of the three study groups. Four patients withdrew consent after randomization (all CG), leaving 61 patients for intention-to-treat analysis (VT *n* = 18; RT *n* = 22; CG *n* = 21). Eight subjects retired early from the study: three due to non-intervention related injuries, four did not respond to invitations for follow-up measurements, and one due to severe chronic illness (breast cancer). Figure 2 shows the detailed CONSORT flow diagram of the study. In seven patients (VT *n* = 2; RT *n* = 3; CG *n* = 2), the final T-scores had to be implemented due to loss of follow-up with the data carried forward method. The mean number of exercise sessions in the VT was *n* = 93 (± 7) and in the RT *n* = 89 (± 7), with an achievable maximum of 104 sessions. The leisure time activity level was assessed for each patient at baseline using the IPAQ questionnaire (version 2005). There were no baseline differences in the metabolic equivalent-minutes per week between groups (*p* = 0.154). Intensity categories (low, moderate, high) were formed according to the IPAQ manual (version 2005) and presented for each group in the supplemental material 2. All baseline characteristics of the participants are presented in Table 1.

**Table 1:** Baseline characteristics of patients by group

	VT (*n* = 18)	RT (*n* = 22)	CG (*n* = 21)	*P* value
Age [years], mean (SD)	56.1 (5.1)	62.8 (6.8)	58.7 (8.2)	0.011^1^
Body mass index [kg/m^2^], mean (SD)	21.6 (2.1)	25.5 (4.0)	24.0 (3.7)	0.003^1^
Osteopenia (%), total hip left, T-score between − 1.0 and − 2.5	66.7	45.5	50.0	0.382^2^
Osteopenia (%), lumbar spine, T-score between − 1.0 and − 2.5	83.3	90.5	90.5	0.732^2^
T-score total hip left, mean (SD)	− 1.16 (0.58)	− 0.84 (0.70)	− 0.92 (0.59)	0.364^3^
T-score lumbar spine, mean (SD)	− 1.81 (0.86)	− 1.77 (0.52)	− 1.86 (0.54)	0.585^3^
EQ-VAS, mean (SD)	85.3 (10.5)	75.0 (15.6)	78.5 (15.2)	0.095^3^
EQ Index, mean (SD)	0.94 (0.07)	0.92 (0.08)	0.89 (0.17)	0.290^3^
Isokinetic strength (60°/s) extensors [Nm], mean (SD)	168.5 (28.3)	122.3 (34.6)	141.3 (43.6)	0.001^3^
Isokinetic strength (60°/s) flexors [Nm], mean (SD)	93.5 (13.0)	74.4 (23.0)	90.0 (32.9)	0.017^3^
Isokinetic strength (240°/s) extensors [Nm], mean (SD)	90.7 (16.8)	65.7 (19.5)	78.2 (24.0)	0.003^3^
Isokinetic strength (240°/s) flexors [Nm], mean (SD)	68.7 (12.7)	53.1 (18.5)	62.6 (19.7)	0.027^3^
Balance test [*n*], mean (SD)	8.2 (3.9)	6.0 (3.7)	7.1 (4.7)	0.030^3^
MET-minutes/week, mean (SD)	1272.3 (1159.4) *n* = 15	1400.1 (956.7) *n* = 19	2261.7 (2293.3) *n* = 17	0.154^1^

*VT* vibration training group, *RT* resistance training group, *CG* control group, *SD* standard deviation, *VAS* visual analogue scale, *MET* metabolic equivalent

^1^One-way ANOVA

^2^Pearson chi-square test

^3^Kruskal–Wallis test

### Follow-up results

#### Primary endpoint

**Fig. 3:**
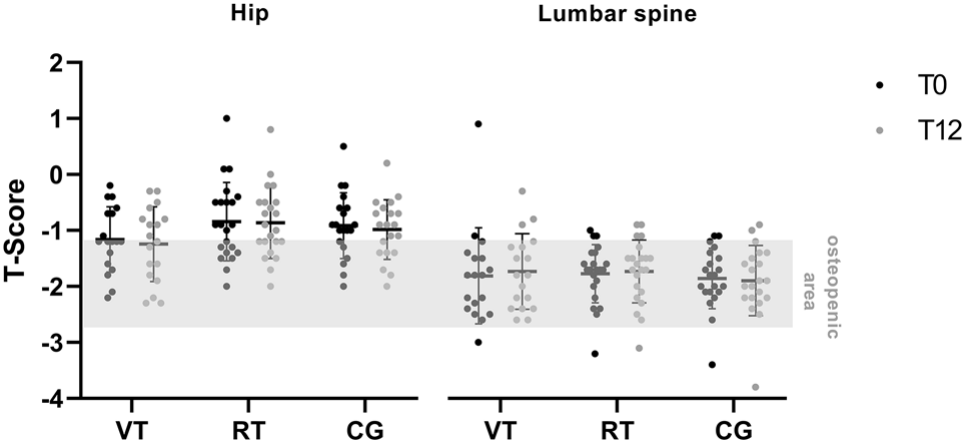
Bone mineral density of the left hip and lumbar spine before (T0) and at the end of intervention (T12) in the vibration training (VT) group, the resistance training (RT) group, and the control group (CG). Individual values are presented together with means ± SD according to the intention-to-treat analysis

Twelve months of WBVT or resistance training did not demonstrate any statically significant effect on BMD (T-score) compared with the CG. The results from the per-protocol and sensitivity analysis were similar to those obtained in the intention-to-treat analysis (Table 2; Fig. 3). Furthermore, no significant changes were seen in the left-sided hip or lumbar spine (T-score) in any group (Table 3). In addition, we analyzed the BMD of the femoral neck as an important subregion of the hip. However, there were no significant changes in any group after the 12-month intervention period in T-score values: VT from − 1.77 ± 0.67 to − 1.74 ± 0.66 (*p* = 0.653); RT from − 1.54 ± 0.66 to − 1.62 ± 0.64 (*p* = 0.069); CG from − 1.63 ± 0.53 to − 1.68 ± 0.66 (*p* = 0.382).

**Table 2:** Between-groups analyses of baseline to 12 month follow-up T-score delta values

	Number of participants (VT/RT/CG)	VT	RT	CG	*P* value^1^
T-score total hip left, mean differences (SD)^2^	18/22/20	0.08 (0.32)	0.02 (0.16)	0.07 (0.19)	0.313
T-score total hip left, mean differences (SD)^3^	16/19/18	0.09 (0.34)	0.04 (0.20)	0.08 (0.20)	0.432
T-score total hip left, mean differences (SD)^4^	16/18/17	0.09 (0.34)	0.02 (0.17)	0.07 (0.20)	0.280
T-score lumbar spine, mean differences (SD)^2^	18/22/21	− 0.08 (0.38)	− 0.04 (0.27)	0.03 (0.25)	0.165
T-score lumbar spine, mean differences (SD)^3^	16/19/19	− 0.09 (0.40)	− 0.05 (0.29)	0.04 (0.26)	0.177
T-score lumbar spine, mean differences (SD)^4^	15/18/18	− 0.17 (0.22)	− 0.04 (0.30)	0.02 (0.26)	0.119

*SD* standard deviation, *VT* vibration training group, *RT* resistance training group, *CG* control group

^1^Kruskal–Wallis test to compare the delta values (T0 baseline – T12 end of interventions)

^2^Intention-to-treat analysis

^3^Per-protocol analysis

^4^Sensitive analysis: statistical analysis without outliers

**Table 3:** Within-groups analyses of T-score values from baseline to 12-month follow-up

	T-score total hip left, T0 (SD)	T-score total hip left, T12 (SD)	Mean difference within group (SD)	*P* value^1^
VT (*n* = 16)	− 1.16 (0.62)	− 1.26 (0.71)	0.10 (0.34)	0.292
RT (*n* = 19)	− 0.82 (0.69)	− 0.84 (0.62)	0.02 (0.17)	0.507
CG (*n* = 18)	− 0.96 (0.61)	− 1.03 (0.54)	0.07 (0.20)	0.115
	T-score lumbar spine, T0 mean (SD)	T-score lumbar spine, T12 mean (SD)		
VT (*n* = 16)	− 1.83 (0.91)	− 1.74 (0.72)	− 0.09 (0.40)	0.399
RT (*n* = 19)	− 1.80 (0.54)	− 1.75 (0.58)	− 0.05 (0.29)	0.490
CG (*n* = 19)	− 1.84 (0.56)	− 1.88 (0.66)	0.04 (0.26)	0.494

Mean difference within group: negative values indicate an improvement

*T0* baseline, *T12* end of interventions, *SD* standard deviation, *VT* vibration training group, *RT* resistance training group, *CG* control group

^1^Paired *t* test

#### Secondary endpoints: between group analysis

##### Isokinetic muscle strength

The analysis of isokinetic muscle strength between the groups showed a statistically significant difference in knee extensors, calculated as the mean difference between the baseline (T0) and 6 (T6) months at an angular velocity of 60°/s (*χ*2 = 8.971, *p* = 0.011) and 240°/s (*χ*2 = 16.567, *p* = 0.000) and between the baseline (T0) and 12 (T12) months at an angular velocity of 240°/s (*χ*2 = 6.694, *p* = 0.035) (supplemental material 3). After 6 months, there was a difference in the left knee extensors at an angular velocity of 60°/s between the RT and the CG (*p* = 0.017, *r* = 0.46) (supplemental material 4). An effect was also observed in the left knee extensors at an angle velocity of 240°/s (*p* > 0.000, *r* = 0.66) (supplemental material 4), which was greater in the VT than in the CG (*p* = 0.018, *r* = 0.46) (supplemental material 4). After 12 months, there was a difference in the left knee extensors at an angle velocity of 240°/s between the RT and the CG (*p* = 0.030, *r* = 0.42) (supplemental material 4).

##### Health- related quality of life (EQ-5D)

After 12 months, EQ-VAS in the RT was significantly improved compared to the CG (*p* = 0.013, *r* = 0.48) (supplemental material 3 and 4).

##### Postural control

For the balance test, no differences between the groups were found at any measurement point.

After 15 months of follow-up (T15), there were no statistically significant differences for the secondary endpoints between groups (supplemental material 1).

#### Secondary endpoints: within-group analysis

##### Isokinetic muscle strength

The isokinetic strength measurements showed statistically significant improvement in all groups, with the exception of the knee extensors at an angle velocity of 60°/s in the CG (*χ*^2^ = 7.473, *p* = 0.058) and the knee flexors at an angle velocity of 240°/s in the VT (*χ*^2^ = 6.600, *p* = 0.058) (Table 4).

**Table 4:** Within-groups analyses of secondary endpoints over all measurement times

Outcome measures	T0 median (25^th^–75^th^ percentiles)	T6 median (25^th^–75^th^ percentiles)	T12 median (25^th^–75^th^ percentiles)	T15 median (25^th^–75^th^ percentiles)	Df	Chi-square	*P* value^1^
Isokinetic strength (60°/s) extensors [Nm]							
VT (*n* = 15):	171 (154–185)	179 (167–201)	191 (164–203)	186 (156–202)	3	12.920	0.005
RT (*n* = 19):	112 (102–155)	134 (121–157)	155 (131–175)	142 (121–161)	3	11.635	0.009
CG (*n* = 17):	142 (93–184)	137 (107–171)	153 (120–185)	149 (115–197)	3	7.473	0.058
Isokinetic strength (60°/s) flexors [Nm]							
VT (*n* = 15):	93 (83–102)	105 (90–117)	100 (92–113)	100 (97–117)	3	11.640	0.009
RT (*n* = 19):	71 (62–91)	85 (69–97)	92 (80–106)	94 (80–106)	3	32.048	> 0.000
CG (*n* = 17):	85 (55–119)	97 (66–119)	93 (69–128)	101 (76–132)	3	8.506	0.037
Isokinetic strength (240°/s) extensors [Nm]							
VT (*n* = 15):	91 (77–105)	100 (88–112)	99 (88–109)	100 (94–109)	3	11.400	0.010
RT (*n* = 19):	66 (53–77)	83 (65–94)	83 (73–92)	84 (69–88)	3	20.116	> 0.000
CG (*n* = 17):	82 (56–99)	78 (60–96)	87 (68–107)	94 (76–106)	3	15.071	0.002
Isokinetic strength (240°/s) flexors [Nm]							
VT (*n* = 15):	64 (59–81)	75 (62–82)	73 (71–81)	78 (66–81)	3	6.600	0.058
RT (*n* = 19):	54 (43–64)	59 (52–77)	63 (51–78)	65 (53–76)	3	20.495	> 0.000
CG (*n* = 17):	70 (43–79)	63 (50–84)	69 (48–89)	74 (57–87)	3	13.024	0.005
EQ-VAS							
VT (*n* = 14):	88 (78–91)	90 (80–91)	85 (80–91)	90 (80–96)	3	0.559	0.906
RT (*n* = 17):	80 (70–90)	80 (80–90)	85 (80–90)	90 (78–91)	3	8.165	0.043
CG (*n* = 16):	80 (73–89)	80 (73–89)	80 (70–90)	80 (73–90)	3	6.062	0.109
EQ Index							
VT (*n* = 14):	1.00 (0.89–1.00)	1.00 (0.89–1.00)	1.00 (0.89–1.00)	1.00 (0.89–1.00)	3	3.643	0.303
RT (*n* = 17):	0.89 (0.89–1.00)	0.89 (0.89–1.00)	1.00 (0.89–1.00)	1.00 (0.89–1.00)	3	4.948	0.176
CG (*n* = 17):	0.89 (0.89–1.00)	0.89 (0.89–1.00)	0.89 (0.89–1.00)	0.89 (0.89–1.00)	3	1.957	0.581
Balance test [*n*]							
VT (*n* = 15):	6 (5–13)	6 (-14)	13 (7–14)	10 (5–14)	3	6.247	0.100
RT (*n* = 18):	5 (5–8)	6 (5–13)	6 (5–14)	5 (5–13)	3	16.895	0.001
CG (*n* = 17):	5 (5–14)	5 (5–14)	5 (5–14)	5 (5–6)	3	6.507	0.089

*Df* degree of freedom, *VT* vibration training group, *RT* resistance training group, *CG* control group, *T0* baseline, *T6* intermediary, *T12* end of interventions, *T15* follow-up

^1^Friedman test

##### Health-related quality of life (EQ-5D)

The VAS of the EQ-5D was statistically significantly improved only in the RT (*χ*^2^ = 8.165, *p* = 0.043) (Table 4).

##### Postural control

There was a statistically significant difference in the balance test only in the RT (*χ*2 = 16.895, *p* = 0.001) (Table 4). All results of the analysis within the group are presented in Table 4 and supplemental material 5 (post hoc tests).

## Discussion

We set out to assess the effects of WBVT and resistance training on BMD T-scores, muscle strength, postural control, and quality of life in comparison to a CG in postmenopausal, osteopenic women. After the 12-month intervention, neither WBVT nor resistance training showed any statistical benefit for BMD T-score values of the lumber spine and total hip as compared to the CG. With two exceptions (CG: 60°/s extensors, VT: 240°/s flexors), there was a significant improvement in muscle strength in the lower extremity within the three study groups (Table 4). This may possibly be due to getting used to the device, although a period of 6 months passed between the first three tests (T0, T6, and T12) and another 3 months between the end of the study (T12) and follow-up (T15). For this reason, however, it is not usual to perform a completely independent, non-evaluated test run on the isokinetic force measurement system. Despite the significant improvements within the groups (Table 4), there are several significant differences between the two intervention groups (VT and RT) and the CG (supplemental material 4), which in turn point to an even bigger increase in strength through physical training. Nevertheless, it is possible that the strength gain of the two intervention groups was not higher due to the training intensity chosen. It is known from the literature that most successful training protocols are using loads corresponding to 80% of 1RM (Marques et al. 2012). Patients of our RT group trained on average with 70% 1RM which could explain the lower strength increases for this group. The achievement of the necessary intensity is also always influenced by personal factors (e.g., motivation, willingness to do exercise until volitional fatigue, fear of injuries, good/bad previous experience with strength training, etc.) and the supervising team can only have limited influence on this. An improvement in the health-related quality-of-life score and balance was observed only in the RT. These beneficial effects on secondary endpoints were lost 3 months after the intervention period (T15). These findings highlight the importance of maintaining an active lifestyle.

### Comparison with existing literature

The non-superiority of WBVT and resistance training as compared to the CG in maintaining BMD is in line with findings of Liphardt et al. (2015) and Mohammad Rahimi et al. (2020). Using comparable vibration settings but different training positions, Liphardt et al. (2015) observed similar declines in BMD and bone architectural parameters in the vibration and the non-training control groups after 20-month follow-up. T-score values in our study remained constant in all groups, but unlike Liphardt et al. (2015), this was only assessed after 12 months. The intervention period may have been too short to detect any significant differences between groups. In addition, resistance training had no beneficial effects on BMD, which is in contrast to current literature (Howe et al. 2011). The work of Howe et al. (2011) demonstrates that especially high impact activities like running/jumping or resistance training exercises using high weights were successful in increasing BMD of the spine, the total hip, or the femoral neck. Consequently, the selected loads of the RT group in our study (70% 1RM) may have been somewhat too low. Additionally, we expected that the chosen vibration settings would produce the necessary impact loads to the skeletal system for triggering anabolic responses. However, the pure vibration time in the VT group was 6 min, which was lower compared to other successful vibration protocols with 12 min (Verschueren et al. 2004) or 10 min (von Stengel et al. 2011a, b). Even high load strength training programs (6–8 exercise machines, 3 sets of 8 repetitions per machine) have a longer load time of about 7.5–10 min, assuming 8 repetitions are performed in 25 s. The lower effective training time could therefore be a further explanation for the lack of improvements in this group. Continuous increases in knee extensor strength were observed in the VT, which is consistent with the current literature (Verschueren et al. 2004; Von Stengel et al. 2011a, b). Of note, our findings demonstrate that participants in the RT and the CG showed improvements for the knee flexors, too. In the RT, study participants showed an almost continuous improvement of knee extensor as well as flexor strength (supplemental material 5). This is in line with the existing literature (Siegrist et al. 2006; Verschueren et al. 2004). Heavy exercise is less attractive in older adults with possible negative impact on long-term adherence, and might expose them to an increased risk of injuries (Von Stengel et al. 2011a, b). Nevertheless, the training settings applied seem to be adequate in postmenopausal women for increasing lower extremity strength without decreasing compliance. This is also supported by the low number of dropouts (RT *n* = 3) during the study period. However, small improvements in knee flexor strength were also observed for the CG, ranging from 4 to 16 Nm. These improvements are probably more related to learning effects than to strength gains. Therefore, these variables have to be interpreted with caution.

Increases in postural control were exclusively found in the RT, possibly due to the 10–15 min balance training. The sensorimotor input via vibration training in our study seems insufficient to improve balance, which is in contrast to a previous report of 8 months of WBVT, showing a significant difference in balance skills, determined by the blind flamingo test (Gusi et al. 2006). In addition, a meta-analysis and systematic review by Ma et al. (2016) reported improvement of fall related factors (e.g., isometric strength, balance, and fall rate) through WBVT. Since we did not record the number of falls, we cannot make any assumptions about the effects that our interventions might have had.

Health-related quality of life assessed by EQ-5D-3L showed increased solely for the RT but only after 12 months of training. It is well known that prolonged physical activity positively affects self-efficacy, self-esteem, and quality of life (Bize et al. 2007).

### Study strengths and limitations

Our study has several strengths and some limitations. All study participants were treated and tested in a 12-month RCT at one institution by specially trained personnel. The compliance was high, indicating that the exercise program was attractive. No adverse events related to the interventions were reported, indicating that WBVT and resistance training are safe treatment options in postmenopausal osteopenic women.

The isokinetic muscle strength significantly increased within all three study groups. This effect was not present when comparing mean differences between the intervention and control group. This may at least partly be due to a Hawthorne effect in the CG mitigating the success of the intervention.

There were baseline differences in age and muscle strength between groups. Patients of the RT group were significantly older and weaker which could have influenced a possible training effect. A study period of 12 months might be too short to induce measurable and meaningful effects of WBVT or resistance training on BMD as compared to our CG. Based on our results and existing evidence, an intervention period of at least 24 months is recommended. Unfortunately, we did not have full access to information on patients’ medication, and therefore, we cannot exclude that some patients might have taken vitamin D supplementation beyond the standard dosage of 800 IE per day.

Furthermore, we are aware that the EuroQol tool (EQ-5D-3L) has its limitations concerning the assessment of health-related quality of life.

## Conclusion

In conclusion, WBVT or resistance training did not lead to significant improvement in BMD (T-score) in postmenopausal osteopenic women after 12 months of controlled intervention compared to physically inactive controls. Muscle strength was improved in the VT and the RT, but improvements were also seen in the CG. In summary, there is limited evidence to determine training intensity and duration of WBVT in postmenopausal women with osteopenia when the treatment goal is defined as an increase in BMD. Nevertheless, health-related quality of life and postural control can be improved by resistance training, which is of clinical relevance for postmenopausal osteopenic women strengthening the importance of regular loading of the musculoskeletal system.

## Supplementary Information

Below is the link to the electronic supplementary material.

Supplemental material 1: Training program of the vibration and resistance training groups. Supplementary file1 (PDF 91 KB)

Supplemental material 2: Percentage of intensity of leisure activities per group at baseline. Supplementary file2 (PDF 83 KB)

Supplemental material 3: Secondary endpoints between groups. Supplementary file3 (PDF 437 KB)

Supplemental material 4: Secondary endpoints: post hoc between groups. Supplementary file4 (PDF 426 KB)

Supplemental material 5: Secondary endpoints: post hoc within groups. Supplementary file5 (PDF 429 KB)

## Abbreviations

A: Amplitude
BMD: Bone mineral density
CG: Control group
Df: Degree of freedom
DMM/kg: Relative peak torque to body mass
F: Frequency
G: Gravity (9.80665 m/s2)
GGT: “Gleichgewichtstest”/ balance test
Hz: Hertz
MET: Metabolic equivalent
N: Number
r: Pearson correlation coefficient
RM: Repetition maximum
RT: Resistance training group
SD: Standard deviation
T0: Baseline
T6: After 6 month interventions
T12: After 12 month interventions
T15: Follow-up after 15 months after start of interventions
VAS: Visual analogue scale
VT: Vibration training group
WBVT: Whole body vibration training
χ^2^: Chi-square
