# Supplementary material for: Effects of whole body vibration in postmenopausal osteopenic women on bone mineral density, muscle strength, postural control and quality of life: the T-bone randomized trial
Source: Eur J Appl Physiol. 2022 Jul 21;122(11):2331–42. doi: 10.1007/s00421-022-05010-5 (PMC9560973; doi:10.1007/s00421-022-05010-5)
Supplement: Supplementary file 1 — Supplemental material 1: Training program of the vibration and resistance training groups. Supplementary file1 (PDF 91 KB) [file 421_2022_5010_MOESM1_ESM.pdf]

Whole body vibration training twice a week fo 12 months

Before vibration training: 10 minutes warm up on a cyclic ergometer

Phase	Exercises during vibration	Vibration settings	Training session	Progression criteria
1	static squats (about 30° knee angle) with or without holding on to the handrail of the vibration device	3 bouts of 1minute (18Hz and 2mm Amplitude) – 6 bouts of 1minute (20Hz and 2mm Amplitude) during the first three weeks	3 -6 bouts of static squats without upper body movement	Hands free standing possible<br>→ Phase 2
2	+ static squats with different upper body exercises (e.g. lifting dummbells, using swing stick)	6 bouts of 1minute (20Hz and 2mm Amplitude) 1 minute rest between sets	3 bouts of static squats without upper body movement<br>+<br>3 bouts of static squats with upper body exercises	The ability to perform dynamic squats without falling for 1 minute<br>→ Phase 3
3	+ dynamic squats	6 bouts of 1minute (20Hz and 2mm Amplitude) 1 minute rest between sets	2 bouts of static squats without upper body movement<br>+<br>2 bouts of static squats with upper body exercises<br>+<br>2 bouts of dynamic squats	

After vibration: stretching of the leg, hip, and lumbar muscles as cool down

Resistance training twice a week fo 12 months

Before resistance training:
10 minutes warm up on a cyclic ergometer
↓
balance exercises for 10-15 minutes (e.g. wobble boards)

↓

Week	Number of series and repetitions	Intensity	Strength devices
0-8	3 x 20 repetitions	50-60% 1RM	leg press, leg abduction, leg flexion, leg extension, latissimus machine and pulley
8-12	3 x 15-20 repetitions	60% 1RM	leg press, leg abduction, leg flexion, leg extension, latissimus machine and pulley
12+	3x 10-15 repetitions	70% 1RM	leg press, leg abduction, leg flexion, leg extension, latissimus machine and pulley

In manuscript
European Journal of Applied Physiology
Effects of whole body vibration in postmenopausal osteopenic women on bone mineral density, muscle strength, postural control and quality of life: The T-Bone randomized trial.
Kienberger Yvonne* 1, Sassmann Robert* 1, Rieder Florian 1, Johansson Tim 2, Kässmann Helmut 3, Pirich Christian 3, Wicker Anton 1, Niebauer Josef 1,4
1 Institute of Physical Medicine and Rehabilitation, Paracelsus Medical University, Salzburg, Austria
2 Institute of General Practice, Family Medicine and Preventive Medicine, Paracelsus Medical University, Salzburg, Austria
3 University Institute of Nuclear Medicine and Endocrinology, Paracelsus Medical University, Salzburg, Austria
4 University Institute of Sports Medicine, Prevention and Rehabilitation, Paracelsus Medical University, Salzburg, Austria
* shared first authorship
Corresponding author:
Correspondance to R. Sassmann (r.sassmann@salk.at)
