# Supplementary material for: Effects of whole body vibration in postmenopausal osteopenic women on bone mineral density, muscle strength, postural control and quality of life: the T-bone randomized trial
Source: Eur J Appl Physiol. 2022 Jul 21;122(11):2331–42. doi: 10.1007/s00421-022-05010-5 (PMC9560973; doi:10.1007/s00421-022-05010-5)
Supplement: Supplementary file 2 — Supplemental material 2: Percentage of intensity of leisure activities per group at baseline. Supplementary file2 (PDF 83 KB) [file 421_2022_5010_MOESM2_ESM.pdf]

Percentage distribution of the intensity of leisure activities per group at baseline

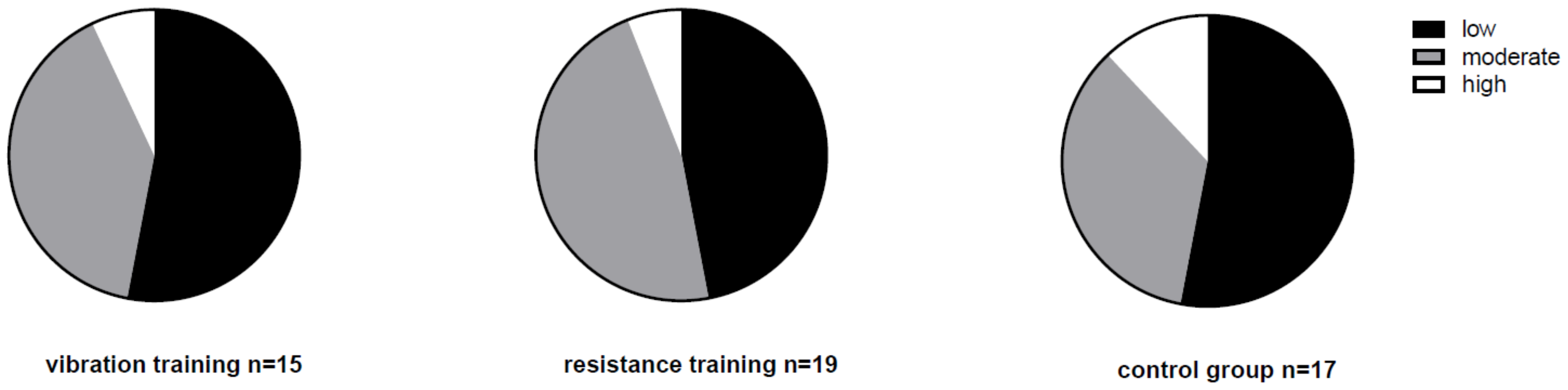

In manuscript
European Journal of Applied Physiology
Effects of whole body vibration in postmenopausal osteopenic women on bone mineral density, muscle strength, postural control and quality of life: The T-Bone randomized trial.
Kienberger Yvonne* 1, Sassmann Robert* 1, Rieder Florian 1, Johansson Tim 2, Kässmann Helmut 3, Pirich Christian 3, Wicker Anton 1, Niebauer Josef 1,4
1 Institute of Physical Medicine and Rehabilitation, Paracelsus Medical University, Salzburg, Austria
2 Institute of General Practice, Family Medicine and Preventive Medicine, Paracelsus Medical University, Salzburg, Austria
3 University Institute of Nuclear Medicine and Endocrinology, Paracelsus Medical University, Salzburg, Austria
4 University Institute of Sports Medicine, Prevention and Rehabilitation, Paracelsus Medical University, Salzburg, Austria
* shared first authorship
Corresponding author:
Correspondance to R. Sassmann (r.sassmann@salk.at)
