# Supplementary material for: Effects of whole body vibration in postmenopausal osteopenic women on bone mineral density, muscle strength, postural control and quality of life: the T-bone randomized trial
Source: Eur J Appl Physiol. 2022 Jul 21;122(11):2331–42. doi: 10.1007/s00421-022-05010-5 (PMC9560973; doi:10.1007/s00421-022-05010-5)
Supplement: Supplementary file 3 — Supplemental material 3: Secondary endpoints between groups. Supplementary file3 (PDF 437 KB) [file 421_2022_5010_MOESM3_ESM.pdf]

Supplemental material 1: Between groups analysis of mean differences secondary endpoints

Variable	Number of participants (VT/RT/CG)	VT	RT	CG	Chi-Square[2]	p-value[2]
**Mean differences (SD) at 6 months[1]**						
EQ-VAS	16/19/17	-2.1 (8.9)	-6.6 (13.0)	0.5 (5.2)	4.193	0.123
EQ Index	16/19/18	-0.01 (0.05)	0.03 (0.15)	0.01 (0.03)	1.242	0.537
Isokinetic strength (60°/s) extensors	18/19/18	-13.8 (15)	-16.2 (20.3)	-0.4 (14.5)	8.971	0.011
Isokinetic strength (60°/s) flexors	18/19/18	-10.7 (11.4)	-13.0 (13.7)	-5.3 (15.0)	3.500	0.174
Isokinetic strength (240°/s) extensors	18/19/18	-9.8 (7.3)	-15.6 (13.0)	-0.9 (9.1)	16.567	0.000
Isokinetic strength (240°/s) flexors	18/19/18	-7.3 (8.2)	-11.7 (9.4)	-3.0 (12.9)	4.925	0.085
Balance test	18/19/18	-0.9 (4.6)	-1.1 (3.3)	-0.5 (2.9)	3.379	0.185
**Mean differences (SD) at 12 months[1]**						
EQ-VAS	14/19/18	-0.9 (7.9)	-7.5 (11.3)	1.8 (10.0)	8.765	0.012
EQ Index	14/19/18	-0.02 (0.06)	0.01 (0.14)	-0.01 (0.07)	0.556	0.757
Isokinetic strength (60°/s) extensors	15/19/18	-17.0 (18.3)	-26.4 (28.0)	-11.0 (23.6)	3.186	0.203
Isokinetic strength (60°/s) flexors	15/19/18	-12.9 (14.5)	-19.9 (15.1)	-8.9 (20.0)	3.833	0.147
Isokinetic strength (240°/s) extensors	15/19/18	-11.6 (8.6)	-17.3 (14.6)	-7.0 (13.6)	6.694	0.035
Isokinetic strength (240°/s) flexors	15/19/18	-8.1 (11.0)	-14.9 (11.9)	-5.5 (13.8)	5.142	0.076
Balance test	15/19/18	-2.53 (5.00)	-2.00 (4.00)	-0.28 (2.95)	5.209	0.074
**Mean differences (SD) at 15 months[1]**						
EQ-VAS	15/18/17	-2.5 (10.1)	-5.0 (18.1)	-1.0 (10.2)	1.355	0.508
EQ Index	15/18/17	-0.01 (0.06)	0.01 (0.16)	-0.01 (0.05)	0.842	0.657
Isokinetic strength (60°/s) extensors	15/19/17	-13.8 (26.3)	-18.6 (20.6)	-11.7 (21.8)	0.921	0.631
Isokinetic strength (60°/s) flexors	15/19/17	-12.9 (17.2)	-21.4 (18.1)	-16.5 (19.4)	1.829	0.401
Isokinetic strength (240°/s) extensors	15/19/17	-11.3 (12.1)	-13.9 (15.6)	-11.6 (13.5)	0.508	0.776
Isokinetic strength (240°/s) flexors	15/19/17	-7.3 (10.4)	-15.3 (13.2)	-10.5 (11.4)	3.878	0.144

Balance test	15/18/17	-1.33 (5.78)	-1.39 (3.76)	1.24 (3.23)	4.308	0.116
Legend: SD= standard deviation, VT= vibration training group, RT= resistance training group, CG= control group<br><br>[1] Mean difference calculated as baseline value minus value at 6, 12 and 15 months. Negative values indicate an improvement.<br>[2] Kruskal-Wallis test						

In manuscript

European Journal of Applied Physiology

Effects of whole body vibration in postmenopausal osteopenic women on bone mineral density, muscle strength, postural control and quality of life: The T-Bone randomized trial.

Kienberger Yvonne* 1, Sassmann Robert* 1, Rieder Florian 1, Johansson Tim 2, Kässmann Helmut 3, Pirich Christian 3, Wicker Anton 1, Niebauer Josef 1,4

1 Institute of Physical Medicine and Rehabilitation, Paracelsus Medical University, Salzburg, Austria

2 Institute of General Practice, Family Medicine and Preventive Medicine, Paracelsus Medical University, Salzburg, Austria

3 University Institute of Nuclear Medicine and Endocrinology, Paracelsus Medical University, Salzburg, Austria

4 University Institute of Sports Medicine, Prevention and Rehabilitation, Paracelsus Medical University, Salzburg, Austria

* shared first authorship

Corresponding author:

Correspondance to R. Sassmann (r.sassmann@salk.at)
