# Supplementary material for: Effects of whole body vibration in postmenopausal osteopenic women on bone mineral density, muscle strength, postural control and quality of life: the T-bone randomized trial
Source: Eur J Appl Physiol. 2022 Jul 21;122(11):2331–42. doi: 10.1007/s00421-022-05010-5 (PMC9560973; doi:10.1007/s00421-022-05010-5)
Supplement: Supplementary file 4 — Supplemental material 4: Secondary endpoints: post hoc between groups. Supplementary file4 (PDF 426 KB) [file 421_2022_5010_MOESM4_ESM.pdf]

Supplemental material 2: Post hoc tests secondary endpoints (pairwise comparison)

		Z	Effect size[1]	p-value[2]
**Isokinetic strength (60°/s) extensors (mean difference at 6 months)**				
RT (n=19)	CG (n=18)	2.769	0.46	0.017
**Isokinetic strength (240°/s) extensors (mean difference at 6 months)**				
RT (n=19)	CG (n=18)	3.991	0.66	0.000
VT (n=18)	CG (n=18)	2.741	0.46	0.018
**EQ-VAS (mean difference at 12 months)**				
RT (n=18)	CG (n=17)	2.848	0.48	0.013
**Isokinetic strength (240°/s) extensors (mean difference at 12 months)**				
RT (n=19)	CG (n=18)	2.580	0.42	0.030
Legend: VT= vibration training group, RT= resistance training group, CG= control group<br><br>[1] Effect size calculated is reported (Cohen 1992), indicating a small (>0.10), moderate >(0.30), or strong effect (>0.50).<br>[2] Dunn-Bonferroni tests. Significance values have been adjusted by the Bonferroni correction for multiple tests.				

In manuscript

European Journal of Applied Physiology

Effects of whole body vibration in postmenopausal osteopenic women on bone mineral density, muscle strength, postural control and quality of life: The T-Bone randomized trial.

Kienberger Yvonne* 1, Sassmann Robert* 1, Rieder Florian 1, Johansson Tim 2, Kässmann Helmut 3, Pirich Christian 3, Wicker Anton 1, Niebauer Josef 1,4

1 Institute of Physical Medicine and Rehabilitation, Paracelsus Medical University, Salzburg, Austria

2 Institute of General Practice, Family Medicine and Preventive Medicine, Paracelsus Medical University, Salzburg, Austria

3 University Institute of Nuclear Medicine and Endocrinology, Paracelsus Medical University, Salzburg, Austria

4 University Institute of Sports Medicine, Prevention and Rehabilitation, Paracelsus Medical University, Salzburg, Austria

* shared first authorship

Corresponding author:

Correspondance to R. Sassmann (r.sassmann@salk.at)
