# Supplementary material for: Effects of whole body vibration in postmenopausal osteopenic women on bone mineral density, muscle strength, postural control and quality of life: the T-bone randomized trial
Source: Eur J Appl Physiol. 2022 Jul 21;122(11):2331–42. doi: 10.1007/s00421-022-05010-5 (PMC9560973; doi:10.1007/s00421-022-05010-5)
Supplement: Supplementary file 5 — Supplemental material 5: Secondary endpoints: post hoc within groups. Supplementary file5 (PDF 429 KB) [file 421_2022_5010_MOESM5_ESM.pdf]

Supplemental material 3: Post hoc tests (pairwise comparisons) secondary endpoints

		Z	Effect size[1]	p-value[2]
**VT (n=15):**				
Isokinetic strength (60°/s) flexors, T0	Isokinetic strength (60°/s) flexors, T12	-3.111	0.80	0.011
Isokinetic strength (60°/s) extensors, T0	Isokinetic strength (60°/s) extensors, T6	-2.828	0.73	0.028
Isokinetic strength (60°/s) extensors, T0	Isokinetic strength (60°/s) extensors, T12	-3.111	0.80	0.011
Isokinetic strength (60°/s) extensors, T0	Isokinetic strength (60°/s) extensors, T15	-2.828	0.73	0.028
Isokinetic strength (240°/s) extensors, T0	Isokinetic strength (240°/s) extensors, T12	-2.970	0.77	0.018
Isokinetic strength (240°/s) extensors, T0	Isokinetic strength (240°/s) extensors, T15	-2.687	0.70	0.043
**RT (n=19):**				
Isokinetic strength (60°/s) flexors, T0	Isokinetic strength (60°/s) flexors, T12	-4.901	1.12	>0.000
Isokinetic strength (60°/s) flexors, T0	Isokinetic strength (60°/s) flexors, T15	-4.586	1.05	>0.000
Isokinetic strength (60°/s) flexors, T6	Isokinetic strength (60°/s) flexors, T12	-2.827	0.65	0.028
Isokinetic strength (60°/s) extensors, T0	Isokinetic strength (60°/s) extensors, T12	-3.141	0.72	0.010
Isokinetic strength (60°/s) extensors, T0	Isokinetic strength (60°/s) extensors, T15	-2.702	0.62	0.041
Isokinetic strength (240°/s) extensors, T0	Isokinetic strength (240°/s) extensors, T6	-3.770	0.86	0.001
Isokinetic strength (240°/s) extensors, T0	Isokinetic strength (240°/s) extensors, T12	-3.895	0.89	0.001
Isokinetic strength (240°/s) extensors, T0	Isokinetic strength (240°/s) extensors, T15	-3.141	0.72	0.010
Isokinetic strength (240°/s) flexors, T0	Isokinetic strength (240°/s) flexors, T12	-3.644	0.84	0.002
Isokinetic strength (240°/s) flexors, T0	Isokinetic strength (240°/s) flexors, T15	-4.147	0.95	>0.000
EQ-VAS, T0	EQ-VAS, T12	-2.125	0.49	0.201[3]
Balance test, T0	Balance test, T12	-2.840	0.65	0.027
**CG (n=17):**				
Isokinetic strength (60°/s) flexors, T0	Isokinetic strength (60°/s) flexors, T15	-2.657	0.64	0.047
Isokinetic strength (240°/s) extensors, T0	Isokinetic strength (240°/s) extensors, T15	-3.122	0.76	0.011
Isokinetic strength (240°/s) extensors, T6	Isokinetic strength (240°/s) extensors, T15	-3.122	0.76	0.011
Isokinetic strength (240°/s) flexors, T0	Isokinetic strength (240°/s) flexors, T15	-3.188	0.77	0.009
Isokinetic strength (240°/s) flexors, T6	Isokinetic strength (240°/s) flexors, T15	-3.055	0.74	0.013

Legend: VT= vibration training group, RT= resistance training group, CG= control group, T0= baseline, T6= intermediary, T12= end of interventions, T15= follow up

[1] Effect size calculated is reported (Cohen 1992), indicating a small (>0.10), moderate >(0.30), or strong effect (>0.50).
[2] Dunn-Bonferroni tests. Significance values have been adjusted by the Bonferroni correction for multiple tests.
[3] P-value is 0.034 when not adjusted by the Bonferroni correction for multiple tests.

In manuscript

European Journal of Applied Physiology

Effects of whole body vibration in postmenopausal osteopenic women on bone mineral density, muscle strength, postural control and quality of life: The T-Bone randomized trial.

Kienberger Yvonne* 1, Sassmann Robert* 1, Rieder Florian 1, Johansson Tim 2, Kässmann Helmut 3, Pirich Christian 3, Wicker Anton 1, Niebauer Josef 1,4

1 Institute of Physical Medicine and Rehabilitation, Paracelsus Medical University, Salzburg, Austria

2 Institute of General Practice, Family Medicine and Preventive Medicine, Paracelsus Medical University, Salzburg, Austria

3 University Institute of Nuclear Medicine and Endocrinology, Paracelsus Medical University, Salzburg, Austria

4 University Institute of Sports Medicine, Prevention and Rehabilitation, Paracelsus Medical University, Salzburg, Austria

* shared first authorship

Corresponding author:

Correspondance to R. Sassmann (r.sassmann@salk.at)
